# Supplementary material for: Mental health services implementation in Colombia–A systematic review
Source: PLOS Glob Public Health. 2023 Dec 6;3(12):e0001565. doi: 10.1371/journal.pgph.0001565 (PMC10699609; doi:10.1371/journal.pgph.0001565)
Supplement: S2 Appendix — (DOCX) [file pgph.0001565.s004.docx]

**Supplementary Data
*S2 Appendix.*** MMAT scores

|  | | **SCREENING QUESTIONS** | | **1. QUALITATIVE STUDIES** | | | | | **4. QUANTITATIVE DESCRIPTIVE STUDIES** | | | | | **Score** |
| --- | --- | --- | --- | --- | --- | --- | --- | --- | --- | --- | --- | --- | --- | --- |
| RefID | Year | S1. Are there clear research questions? | S2. Do the collected data allow to address the research questions? | 1.1. Is the qualitative approach appropriate to answer the research question? | 1.2. Are the qualitative data collection methods adequate to address the research question? | 1.3. Are the findings adequately derived from the data? | 1.4. Is the interpretation of results sufficiently substantiated by data? | 1.5. Is there coherence between qualitative data sources, collection, analysis and interpretation? | 4.1. Is the sampling strategy relevant to address the research question? | 4.2. Is the sample representative of the target population? | 4.3. Are the measurements appropriate? | 4.4. Is the risk of nonresponse bias low? | 4.5. Is the statistical analysis appropriate to answer the research question? |  |
| Barrios Acosta 2017 | 2017 | Yes | Yes | Yes | Yes | Yes | Can't tell | Yes |  |  |  |  |  | **4.3** |
| Borda 2021 | 2021 | Yes | Yes |  |  |  |  |  | No | No | Yes | Yes | Yes | **3.0** |
| Castro 2020 | 2020 | Yes | Yes |  |  |  |  |  | Yes | Yes | Yes | Can't tell | Yes | **4.3** |
| Ceballos 2016 | 2016 | Yes | Yes | Yes | Yes | Yes | Yes | Can't tell |  |  |  |  |  | **4.3** |
| Gomez Restrepo 2018 | 2018 | Yes | Yes |  |  |  |  |  | Yes | Yes | Yes | No | Yes | **4.0** |
| Gonzalez 2019 | 2019 | Yes | Yes |  |  |  |  |  | Yes | Yes | Yes | Can't tell | Yes | **4.3** |
| Hernandez Holguin 2017 | 2017 | Yes | Yes | Yes | Yes | Can't tell | Yes | Yes |  |  |  |  |  | **4.3** |
| Martinez Perez 2020 | 2020 | Yes | Yes |  |  |  |  |  | Can't tell | Yes | Yes | Yes | Yes | **4.3** |
| Mejia Trujillo 2015 | 2015 | Yes | Yes |  |  |  |  |  | Can't tell | No | Yes | Can't tell | Yes | **2.5** |
| Satizabal Reyes 2019 | 2019 | Yes | Can't tell | Yes | Can't tell | Yes | Can't tell | Can't tell |  |  |  |  |  | **2.8** |
| Shannon 2021 | 2021 | Yes | Yes | Yes | Yes | Yes | Yes | Yes |  |  |  |  |  | **5.0** |
| Taborda Zapata 2016 | 2016 | Yes | Can't tell | Yes | Can't tell | Yes | Yes | Can't tell |  |  |  |  |  | **3.5** |
| **Total Score** | | | | | | | | | | | | | | **3.6** |
